# Supplementary material for: Combined IgE neutralization and Bifidobacterium longum supplementation reduces the allergic response in models of food allergy
Source: Nat Commun. 2022 Sep 27;13:5669. doi: 10.1038/s41467-022-33176-1 (PMC9515155; doi:10.1038/s41467-022-33176-1)
Supplement: Supplementary file 1 — Supplementary Information [file 41467_2022_33176_MOESM1_ESM.pdf]

Combined IgE neutralization and *Bifidobacterium longum* supplementation reduces the allergic responses in food allergy models

Seong Beom An[1,2,*], Bo-Gie Yang[3,4,*], Gyeonghui Jang[3], Do-Yeon Kim[3], Jiyoung Kim[3], Sung-Man Oh[3], Nahyun Oh[3], Sanghee Lee[3], Ji-Yeong Moon[5], Jeong-Ah Kim[3], Ji-Hyun Kim[3], Yoo-Jeong Song[3], Hye-Won Hyun[3], Jisoo Kim[3], Kyungwha Lee[3], Dajeong Lee[4], Min-Jung Kwak[4], Byung Kwon Kim[4], Young-Kyu Park[4], Chun-Pyo Hong[3,6], Jung Hwan Kim[7], Hye Seong Lim[7], Min Sook Ryu[5], Hyun-Tak Jin[7], Seung-Woo Lee[1,2], Yoon-Seok Chang[8], Hae-Sim Park[5], Young Chul Sung[1,2,] and Myoung Ho Jang[3,9,]

[1] Division of Integrative Biosciences and Biotechnology, Pohang University of Science and Technology (POSTECH), Pohang, Gyeongbuk, Republic of Korea

[2] Department of Life Sciences, Pohang University of Science and Technology (POSTECH), Pohang, Gyeongbuk, Republic of Korea

[3] Research Institute, GI Innovation Inc., Songpa, Seoul, Republic of Korea

[4] Research Institute, GI Biome Inc., Seongnam, Gyeonggi-do, Republic of Korea

[5] Department of Allergy and Clinical Immunology, Ajou University School of Medicine, Suwon, Republic of Korea

[6] Research Institute, GI Cell Inc., Seongnam, Gyeonggi-do, Republic of Korea

[7] Research Institute, ProGen Inc., Korea Bio Park, Seongnam, Gyeonggi-do, Republic of Korea

[8] Department of Internal Medicine, Seoul National University Bundang Hospital, Seoul National University College of Medicine, Seongnam, Republic of Korea

[9] World Premier International Immunology Frontier Research Center, Osaka University, Suita, Japan

* These authors contributed equally to this work

# address correspondence to

: jangmh@gi-innovation.com, ycsung@postech.ac.kr or yangbg@gi-biome.com

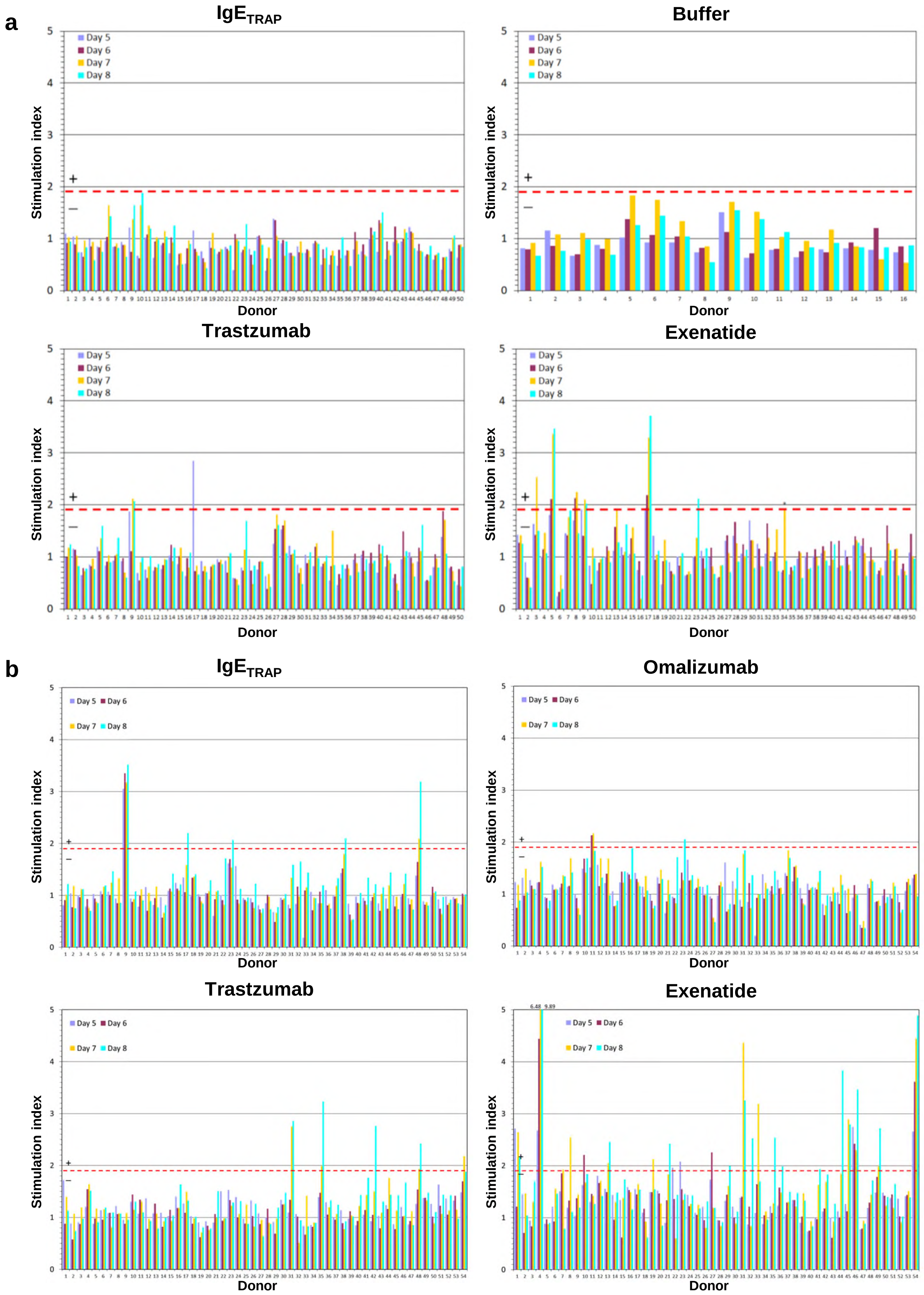

Supplementary Figure 1. T cell proliferation in response to IgE_{TRAP} exposure assessed by EpiScreen assay with T cells from healthy human donors.

PBMCs from at least 50 healthy human donors were depleted of $CD8^+$ T cells. PBMCs were cultured for up to 8 days at 37ºC with 5% CO_2 in the presence of IgE_{TRAP}, buffer (IgE_{TRAP} formulation buffer), omalizumab, trastzumab or exenatide. On days 5, 6, 7 and 8, T cell proliferations were assessed by looking at [^{3}H]-Thymidine incorporation. This experiment was independently performed twice. The first experimental result is shown in **(a)** and the second experimental result is shown in **(b)**. A response was considered positive if the stimulation index (SI) was equal to or greater than 1.9 (SI ≥ 1.90). Red dotted line indicates the SI value of SI 1.9 (SI = 1.9).

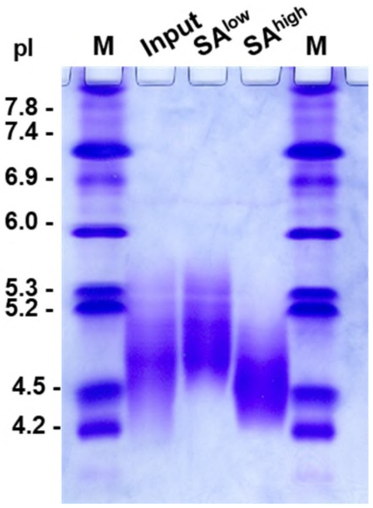

Supplementary Figure 2. Separation of IgE$_{TRAP}$ with high and low sialic acid content by isoelectric focusing.

IgE$_{TRAP}$ with high and low sialic acid content (IgE$_{TRAP}$ SA^{high}, 20.9 mol mol^{-1}; IgE$_{TRAP}$ SA^{low}, 11.4 mol mol^{-1}) were purified using an anion exchange column and separated by isoelectric focusing (IEF). Input refers to the IgE$_{TRAP}$ protein which was not processed for purification step using an anion exchange column. The figure is representative of three independent experiments. Source data are provided as a Source Data file.

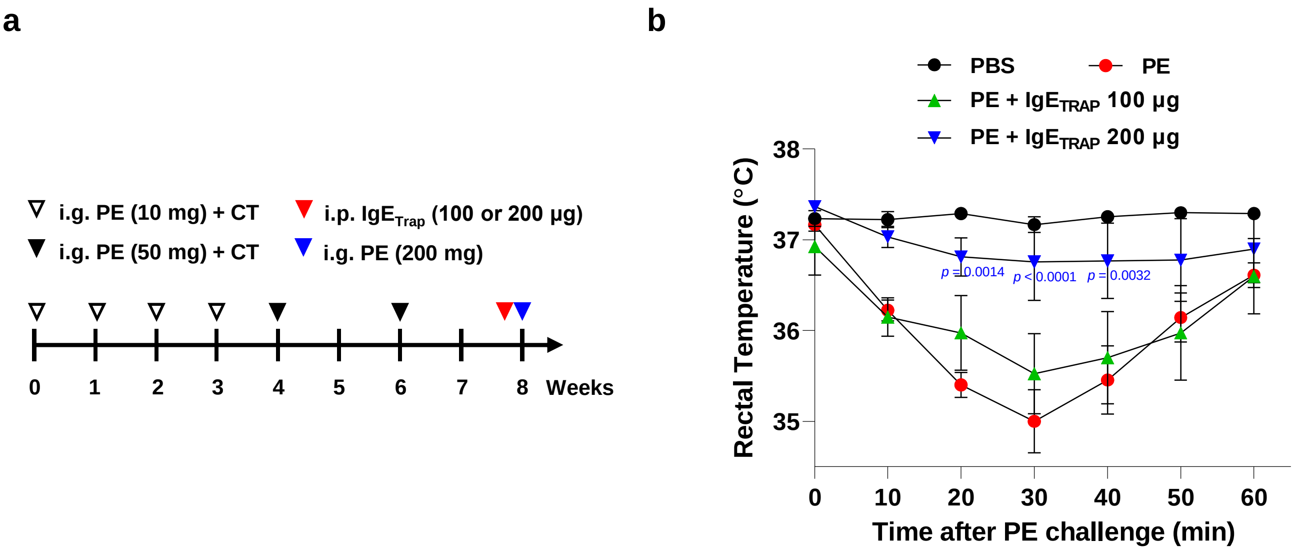

Supplementary Figure 3. Dose-dependent therapeutic effect of IgE$_{TRAP}$ in a peanut-induced systemic anaphylaxis model.

a, Experimental scheme of peanut-induced systemic anaphylaxis induction in mice and administration of IgE$_{TRAP.}$ **b,** Suppression of rectal temperature drop by IgE$_{TRAP}$. Statistical analysis was performed by Two-way repeated ANOVA with Dunnett's multiple comparison with PE group as a control (n = 9 mice in groups of PBS (Black circle), PE (Red circle), and PE + IgE$_{TRAP}$ 200 μg (Blue triangle); 8 mice in PE + IgE$_{TRAP}$ 100 μg group (Green triangle)). Data are presented as means values ± SEM. Specific p-values are indicated in the figure. Source data are provided as a Source Data file.

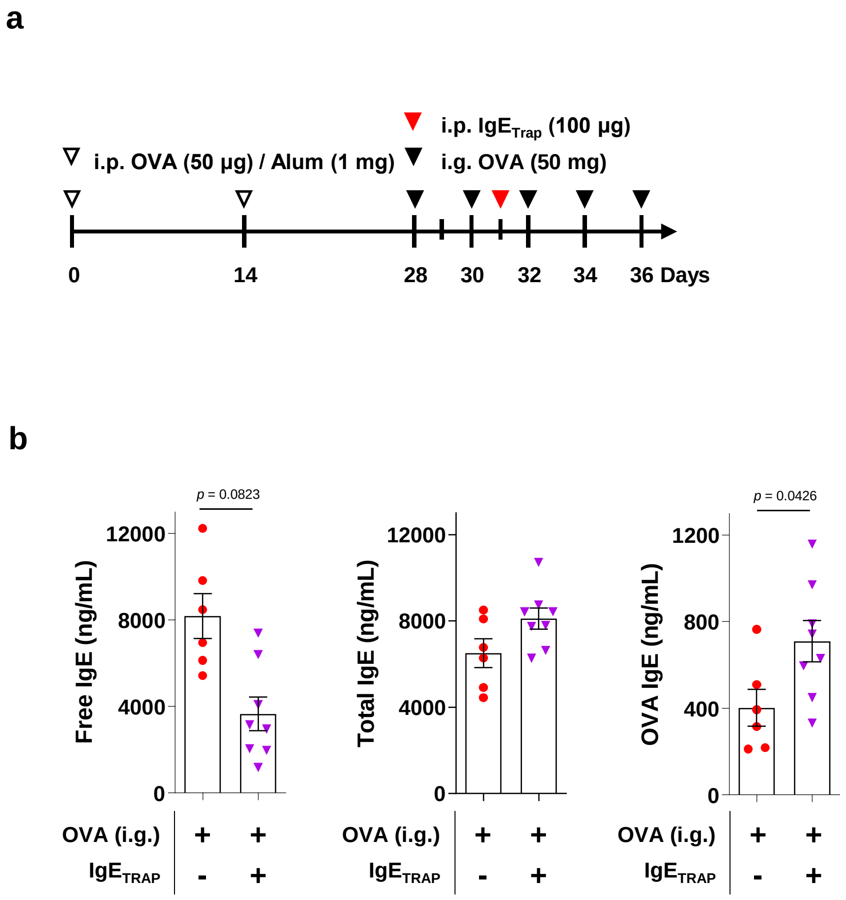

Supplementary Figure 4. Quantification of free IgE, total IgE and OVA-specific IgE before and after IgE$_{TRAP}$ treatment of mice with OVA-induced food allergy.

a, Experimental scheme for food allergy induction and IgE$_{TRAP}$ administration. **b,** Serum levels of free, total, and OVA-specific IgE measured by ELISA (*n* = 6 mice in OVA group (Red circle); 8 mice in OVA + IgE$_{TRAP}$ group (Purple triangle)). Statistical analysis was performed by unpaired two-tailed Student's t-test (b). Data are presented as means values ± SEM. Specific *p*-values are indicated in the figure. Source data are provided as a Source Data file.

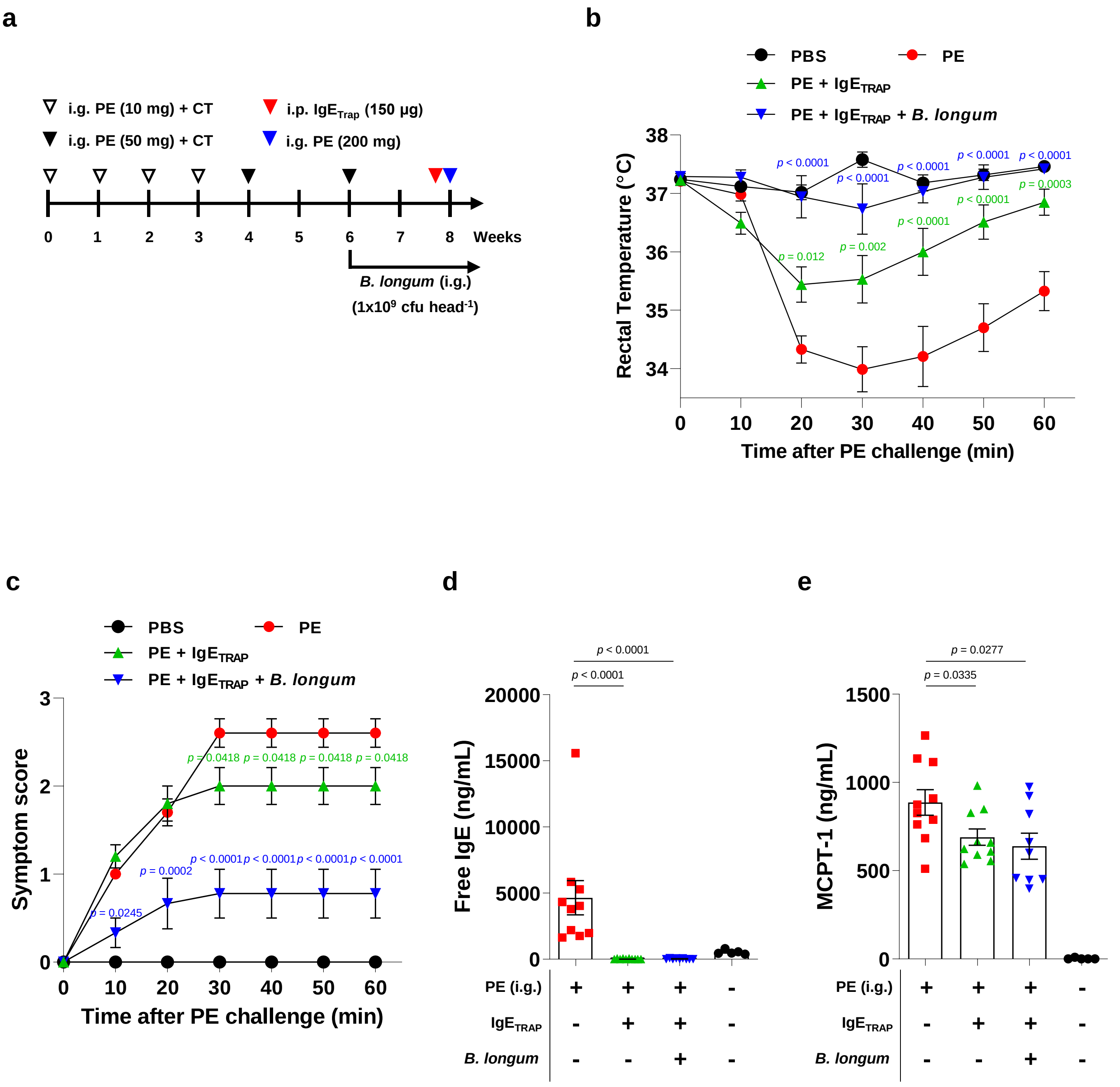

Supplementary Figure 5. Enhancement of the therapeutic effect of IgE$_{TRAP}$ by lower dose *B. longum* administered for shorter period in mice with peanut-induced systemic anaphylaxis.

a, Experimental scheme for peanut food allergy and administration of IgE$_{TRAP}$ and *B. longum*. *B. longum* (1x10^9 cfu head^{-1}) was intragastrically administered to mice daily starting from when the mice received the second administration with 50 mg peanut extract (PE) and cholera toxin (CT). i.p, intraperitoneal; i.g., intragastric. **b and c,** Changes of rectal temperature **(b)** and symptom score **(c)** by IgE$_{TRAP}$ alone and IgE$_{TRAP}$ in combination with *B. longum*. Statistical analysis was performed by Two-way repeated ANOVA with Dunnett's multiple comparison using PE group as a control. **d and e**, Serum levels of Free IgE **(d)** and MCPT-1 **(e)** (n = 5 mice in PBS group (Black circle); 10 mice in groups of PE (Red circle) and PE + IgE$_{TRAP}$ (Green triangle); 9 mice in PE + IgE$_{TRAP}$ + *B. longum* group (Blue triangle)). Statistical analysis was performed by unpaired two-tailed Student's t-test . Data are presented as means values ± SEM. Specific *p*-values are indicated in the figure. Source data are provided as a Source Data file.

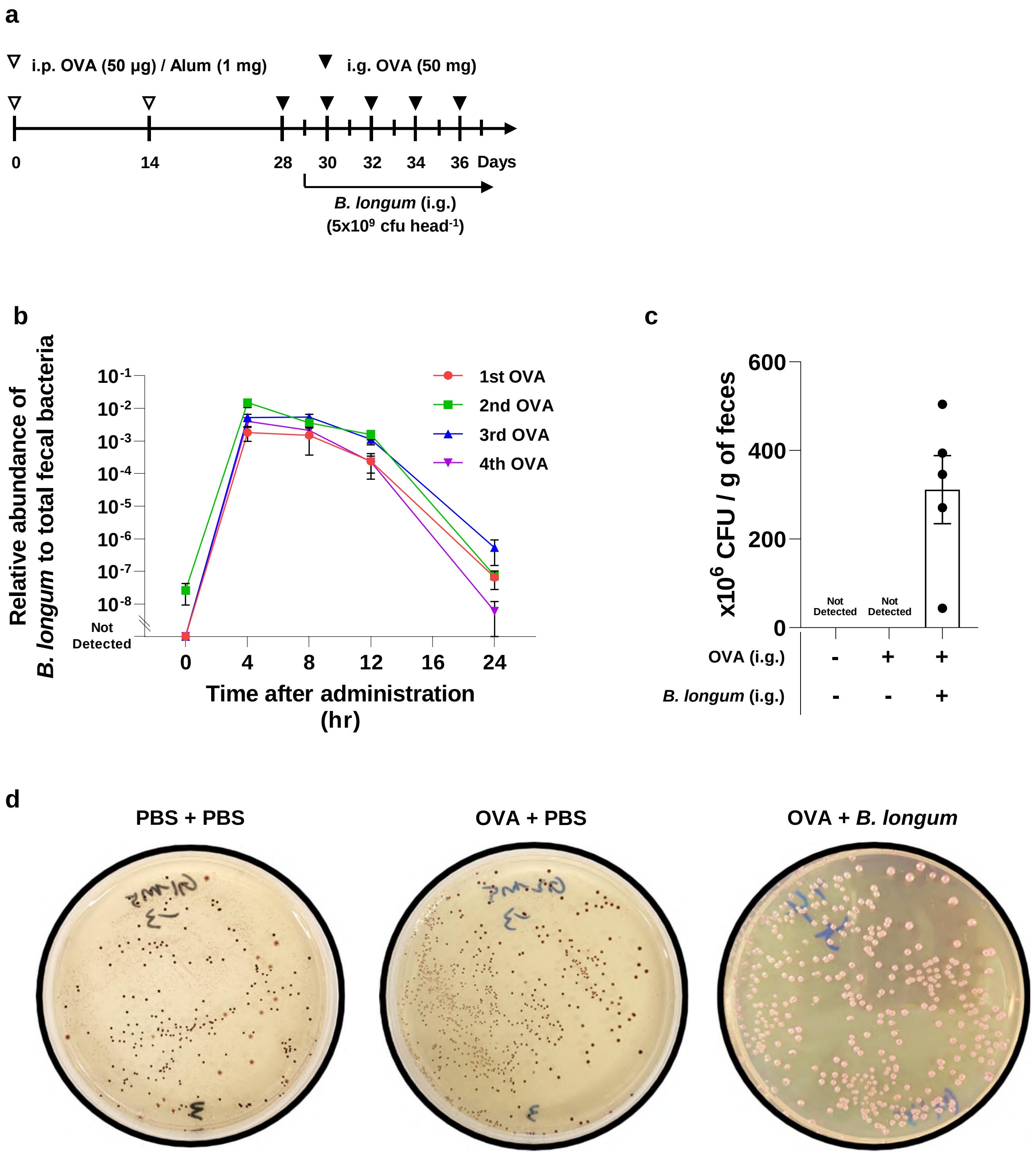

Supplementary Figure 6. Quantification of *B. longum* in feces after its oral administration in an OVA-induced food allergy model.

a, Experimental scheme for oral administration of *B. longum* in an OVA-induced food allergy model. **b,** Relative quantification of *B. longum* in fecal samples. After each oral administration of OVA, relative abundance of *B. longum* among total bacteria was measured over time by 16S rRNA gene-targeted PCR analysis. **c and d,** Quantification of *B. longum* in fecal samples by culture. After the third oral administration of OVA, *B. longum* was recovered from feces obtained 4 hours after the oral administration and the amount of *B. longum* was calculated as colony forming units (CFU) per 1 gram of feces. Quantitative graph **(c)** and representative picture **(d)** of *B. longum* cultured on Bifidus Selective Medium (BSM) agar plates are shown (*n* = 5 mice per group). Data are presented as means values ± SEM. Source data are provided as a Source Data file.

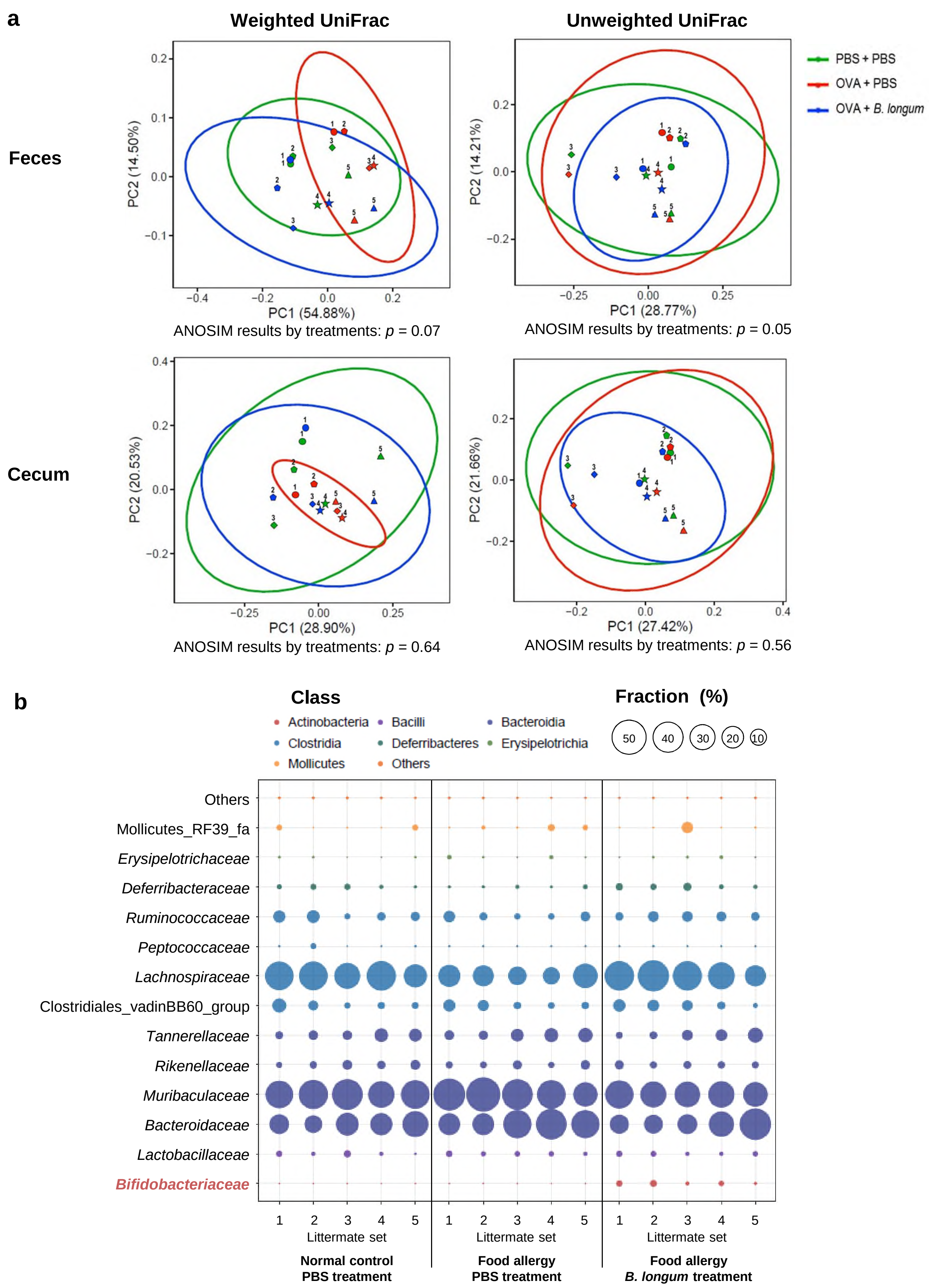

Supplementary Figure 7. Effects of *B. longum* administration on gut microbial community.

a. Analysis of the effect of *B. longum* on the intestinal microbial community. Feces and cecal contents were collected after the last oral administration of *B. longum* and comparison of beta diversity of bacterial communities in the feces and cecal content were analyzed using principal coordinates analysis (PCoA). Weighted and Unweighted UniFrac distance values were calculated by one-way ANOSIM (analysis of similarities) test. The markings with same number and shape are littermates. **b,** Family-level bacterial taxonomic compositions in the feces after the last oral administration of *B. longum*. (n = 5 per group). Specific p-values are indicated in the figure.

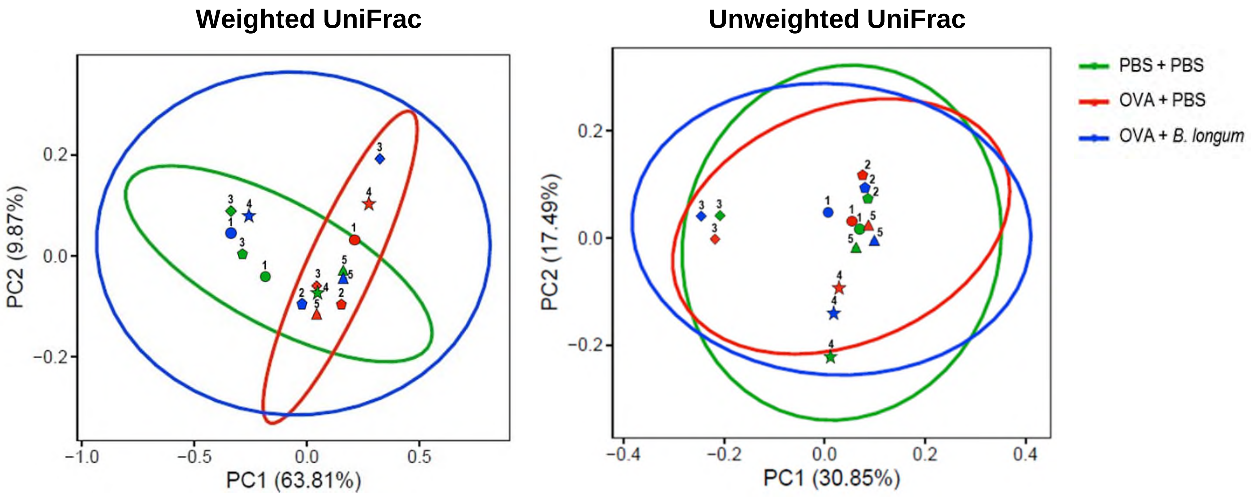

ANOSIM results by treatments: $p = 0.205$
ANOSIM results by littermates: $p = 0.655$

ANOSIM results by treatments: $p = 0.985$
ANOSIM results by littermates: $p < 0.001$, R = 0.799

Supplementary Figure 8. Similarity of gut microbial community between littermates before OVA and *B. longum* treatment.

Feces were collected before the oral administration of OVA and *B. longum*, and the beta diversity of bacterial communities in the feces were compared by principal coordinates analysis (PCoA). Weighted and Unweighted UniFrac distance values were calculated by one-way ANOSIM test. The test subjects identified by the same number and shape of marker were littermates. ($n = 5$ per group). Specific *p*-values and R-values are indicated in the figure.

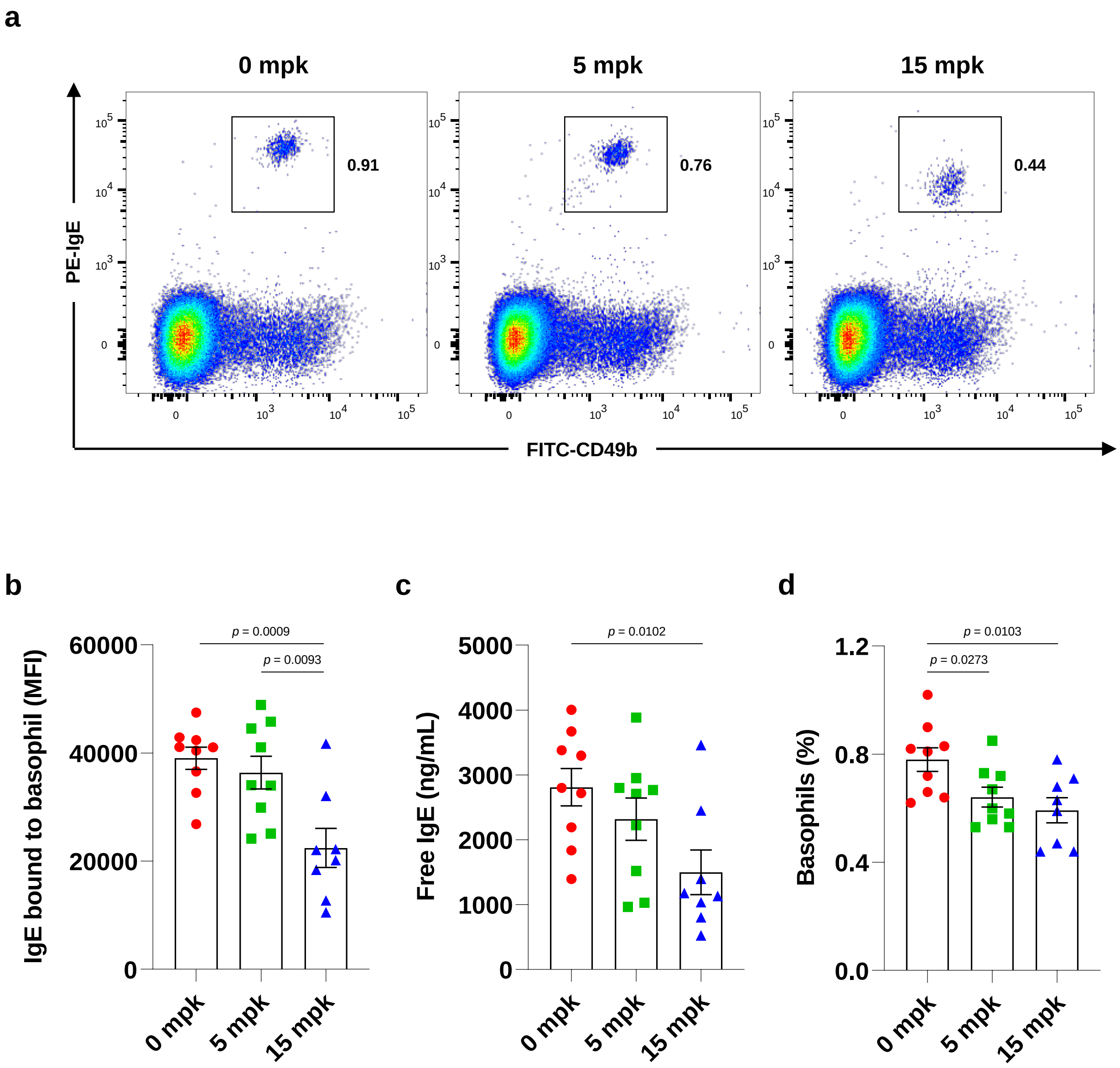

Supplementary Figure 9. Suppressive effect of IgE_{TRAP} on IgE binding to basophils, proliferation of basophil and serum levels of free IgE in an OVA-induced food allergy model.
In OVA-induced food allergy model, 0, 5 and 15 mpk (mg kg^{-1}) of IgE_{TRAP} was intraperitoneally administered a day after the last oral challenge of OVA. Basophils in blood were analyzed by flow cytometry after one week. (n = 9 mice in 0 mpk group (Red circle); 8 mice in 5 mpk group (Green square); 7 mice in 15 mpk group (Blue triangle)). **a,** Representative flow cytometry data. **b-d,** Mean fluorescence intensities (MFI) of IgE bound on basophil **(b)**, serum free IgE levels **(c)** and proportion of basophils in blood **(d)** represented as dot/bar graphs. Statistical analysis was performed by unpaired two-tailed Student's t-test. Data are presented as means values ± SEM. Specific p-values are indicated in the figure. Source data are provided as a Source Data file.

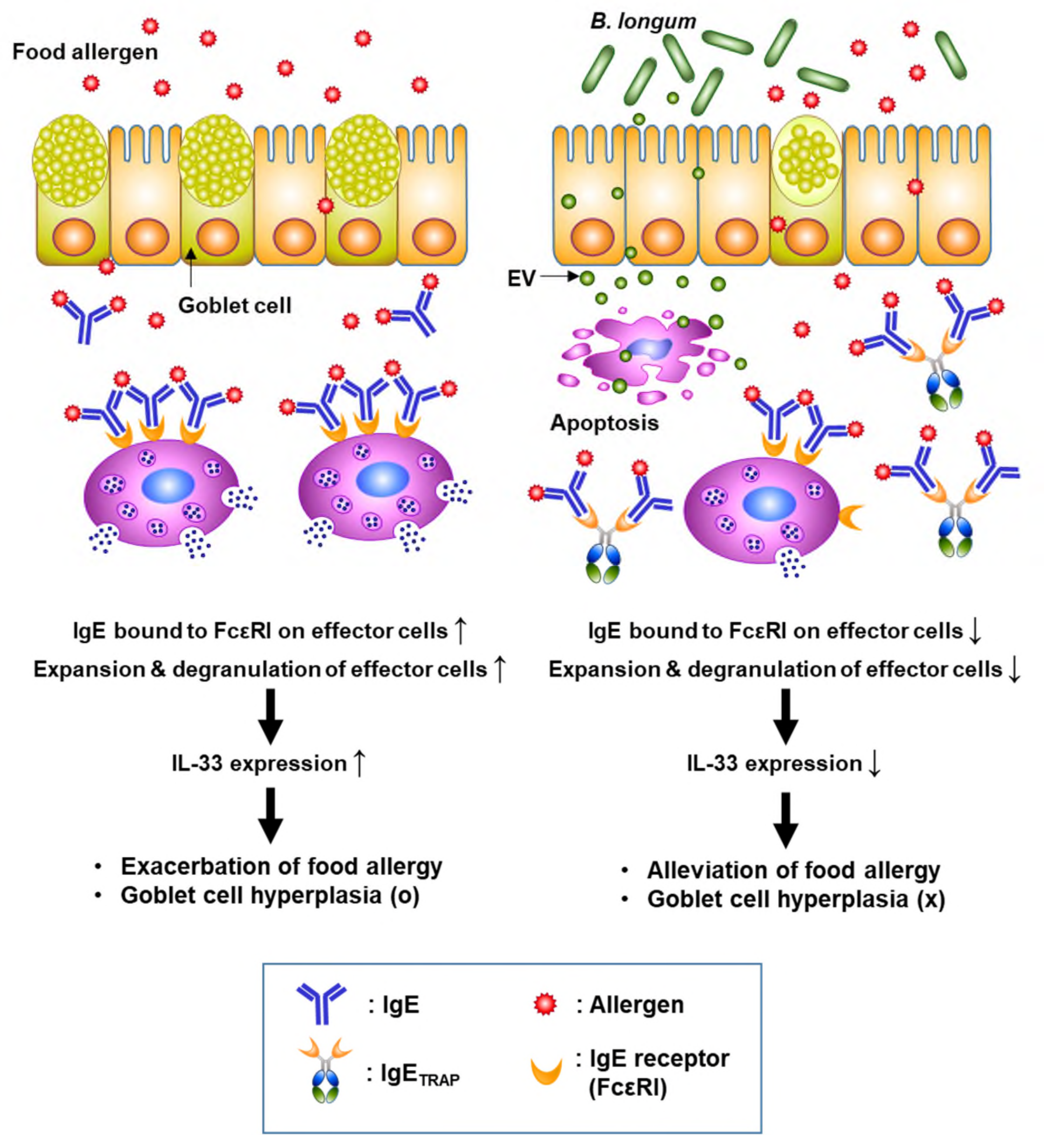

Supplementary Figure 10. Proposed mechanism of food allergy suppression by *B. longum* and IgE$_{TRAP}$ combination therapy.

Ingested food allergens induce activation of effector cells (mast cells and basophils) by binding to IgE and cross-linking the high affinity IgE receptor (FcεRI) on these cells, which then release mediators resulting in a hypersensitivity reaction. *B. longum* induces mast cell apoptosis through extracellular vesicle (EV) secretion, reducing mast cell numbers. A main component of EVs is family 5 extracellular solute-binding protein involved in the induction of mast cell apoptosis. Meanwhile, IgE$_{TRAP}$ suppresses the activation and proliferation of effector cells by blocking IgE binding to FcεRI on effector cells. The numerical reduction of mast cells leads to a reduction in *IL-33* expression, which is greater when IgE$_{TRAP}$ and *B. longum* are given in combination. According to previous reports[S11, S12, S13], mast cells produce IL-33, which worsens food allergy symptoms and goblet cell hyperplasia. Combination therapy with IgE$_{TRAP}$ and *B. longum* effectively alleviates both food allergy symptoms and goblet cell hyperplasia.

**Supplementary Table 1 | EpiScreen assay**			
Sample	Mean SI*	SD	% Response
IgE_{TRAP}	N/A	N/A	0
Buffer (IgE_{TRAP} formulation buffer)	N/A	N/A	0
Trastzumab	2.35	± 0.43	4
Exenatide	2.47	± 0.64	14
Keyhole limpet hemocyanin (KLH)	9.15	± 8.75	90

*Stimulation Index (SI) = mean value of test wells (cpm) / baseline (cpm). The mean SI was calculated from the average of all positive donor responses observed during the entire time course (days 5-8). N/A indicates no data available.

Supplementary Table 2. Changes of gut microbiota at genus level by oral administration of *B. longum*

Taxonomic group (Genus level)	Before *B. longum* treatment	After *B. longum* treatment	
	feces	feces	cecum
Bifidobacterium	0.00	**7.93***	**6.30***
Mucispirillum	-5.16	4.67*	-3.21
Ruminiclostridium_5	-0.96	1.81*	-0.79

The values are coefficients from the generalized linear regression multiplied by 100. Red color indicates positive coefficient values and the intensity of the color is proportional to the magnitude of the value.
Values that significantly different from values before the treatment are marked with asterisk (* $p < 0.05$ & FDR < 0.20)

Supplementary references

S1. Callahan, B. J., McMurdie, P. J., Rosen, M. J., Han, A. W., Johnson, A. J. A., & Holmes, S. P. (2016). DADA2: high-resolution sample inference from Illumina amplicon data. Nature methods, 13(7), 581-583.

S2. Jiang, H., Lei, R., Ding, S. W., & Zhu, S. (2014). Skewer: a fast and accurate adapter trimmer for next-generation sequencing paired-end reads. BMC bioinformatics, 15(1), 1-12.

S3. Schloss PD*, et al.* Introducing mothur: open-source, platform-independent, community-supported software for describing and comparing microbial communities. *Appl Environ Microbiol* **75**, 7537-7541 (2009).

S4. Quast C*, et al.* The SILVA ribosomal RNA gene database project: improved data processing and web-based tools. *Nucleic Acids Res* **41**, D590-596 (2013).

S5. Westcott SL, Schloss PD. OptiClust, an Improved Method for Assigning Amplicon-Based Sequence Data to Operational Taxonomic Units. *mSphere* **2**, (2017).

S6. Lozupone C, Knight R. UniFrac: a new phylogenetic method for comparing microbial communities. *Appl Environ Microbiol* **71**, 8228-8235 (2005).

S7. Mallick H, Rahnavard A, McIver LJ, Ma S, Zhang Y, Nguyen LH, Tickle TL, Weingart G, Ren B, Schwager EH, Chatterjee S, Thompson KN, Wilkinson JE, Subramanian A, Lu Y, Waldron L, Paulson JN, Franzosa EA, Bravo HC, Huttenhower C (2021). Multivariable Association Discovery in Population-scale Meta-omics Studies. PLoS Computational Biology, 17(11):e1009442.

S8. Yoon SH*, et al.* Introducing EzBioCloud: a taxonomically united database of 16S rRNA gene sequences and whole-genome assemblies. *Int J Syst Evol Microbiol* **67**, 1613-1617 (2017).

S9. Junick J, Blaut M. Quantification of human fecal bifidobacterium species by use of quantitative real-time PCR analysis targeting the groEL gene. *Appl Environ Microbiol*. 2012;78(8):2613-2622.

S10. Matsuki T, Watanabe K, Fujimoto J, Kado Y, Takada T, Matsumoto K, Tanaka R. Quantitative PCR with 16S rRNA-gene-targeted species-specific primers for analysis of human intestinal bifidobacteria. Appl Environ Microbiol. 2004 Jan;70(1):167-73.

S11. Galand C, et al. IL-33 promotes food anaphylaxis in epicutaneously sensitized mice by targeting mast cells. J Allergy Clin Immunol 138, 1356-1366 (2016)

S12. Saluja R, Khan M, Church MK, Maurer M. The role of IL-33 and mast cells in allergy and inflammation. Clin Transl Allergy 5, 33 (2015).

S13. Hsu CL, Neilsen CV, Bryce PJ. IL-33 is produced by mast cells and regulates IgE-dependent inflammation. PLoS One 5, e11944 (2010)
