# Supplementary material for: Combined IgE neutralization and Bifidobacterium longum supplementation reduces the allergic response in models of food allergy
Source: Nat Commun. 2022 Sep 27;13:5669. doi: 10.1038/s41467-022-33176-1 (PMC9515155; doi:10.1038/s41467-022-33176-1)
Supplement: Supplementary file 3 — Description of Additional Supplementary Files [file 41467_2022_33176_MOESM3_ESM.pdf]

Description of Additional Supplementary Files

Supplementary Data 1. BLI assay result for the interaction of omalizumab with Fc gamma receptor and C1q

Supplementary Data 2. BLI assay result for the interaction of IgE_{TRAP} with Fc gamma receptor and C1q

Supplementary Data 3. SPR assay result for the interaction of omalizumab and IgE_{TRAP} with IgE

Supplementary Data 4. BLI assay for the interaction of omalizumab and IgE_{TRAP} with $Fc\varepsilon RI\alpha$-IgE complex.
