# Supplementary material for: Combined IgE neutralization and Bifidobacterium longum supplementation reduces the allergic response in models of food allergy
Source: Nat Commun. 2022 Sep 27;13:5669. doi: 10.1038/s41467-022-33176-1 (PMC9515155; doi:10.1038/s41467-022-33176-1)
Supplement: Supplementary file 8 — Reporting Summary [file 41467_2022_33176_MOESM8_ESM.pdf]

nature research

Corresponding author(s): Myoung Ho Jang

Last updated by author(s): Aug 23, 2022

Reporting Summary

Nature Research wishes to improve the reproducibility of the work that we publish. This form provides structure for consistency and transparency in reporting. For further information on Nature Research policies, see our Editorial Policies and the Editorial Policy Checklist.

Statistics

For all statistical analyses, confirm that the following items are present in the figure legend, table legend, main text, or Methods section.

n/a	Confirmed	
☐	☒	The exact sample size (n) for each experimental group/condition, given as a discrete number and unit of measurement
☐	☒	A statement on whether measurements were taken from distinct samples or whether the same sample was measured repeatedly
☐	☒	The statistical test(s) used AND whether they are one- or two-sided<br>*Only common tests should be described solely by name; describe more complex techniques in the Methods section.*
☒	☐	A description of all covariates tested
☐	☒	A description of any assumptions or corrections, such as tests of normality and adjustment for multiple comparisons
☐	☒	A full description of the statistical parameters including central tendency (e.g. means) or other basic estimates (e.g. regression coefficient) AND variation (e.g. standard deviation) or associated estimates of uncertainty (e.g. confidence intervals)
☐	☒	For null hypothesis testing, the test statistic (e.g. F, t, r) with confidence intervals, effect sizes, degrees of freedom and P value noted<br>*Give P values as exact values whenever suitable.*
☒	☐	For Bayesian analysis, information on the choice of priors and Markov chain Monte Carlo settings
☒	☐	For hierarchical and complex designs, identification of the appropriate level for tests and full reporting of outcomes
☒	☐	Estimates of effect sizes (e.g. Cohen's d, Pearson's r), indicating how they were calculated

Our web collection on statistics for biologists contains articles on many of the points above.

Software and code

Policy information about availability of computer code

Data collection	BD FACS Diva (v8.0)
Data analysis	Graphpad Prism (v9.3.1)<br>FlowJo (v10.8.0)<br>Phoenix WinNonlin program (v8.1)<br>R language (v4.0.4)<br>(DADA2 package v1.18.0, Mothur package v1.43.0, MaAsLin2 package v1.7.3, ggplot2 package v3.3.5, ggpubr package v0.4.0)<br>Skewer program (v0.2.1)

For manuscripts utilizing custom algorithms or software that are central to the research but not yet described in published literature, software must be made available to editors and reviewers. We strongly encourage code deposition in a community repository (e.g. GitHub). See the Nature Research guidelines for submitting code & software for further information.

Data

Policy information about availability of data

All manuscripts must include a data availability statement. This statement should provide the following information, where applicable:

- Accession codes, unique identifiers, or web links for publicly available datasets
- A list of figures that have associated raw data
- A description of any restrictions on data availability

The structure of IgETRAP was based on the Protein Data Bank (PDB accession 1F6A and 1ADQ). The 16s rRNA analysis data of fecal and cecal samples generated in this study have been deposited in the National Center for Biotechnology Information (NCBI) database under accession code PRJNA853889. All data generated in this

April 2020

study are provided in the Supplementary Information/Source Data file. Source data are provided with this paper.

Field-specific reporting

Please select the one below that is the best fit for your research. If you are not sure, read the appropriate sections before making your selection.

[x] Life sciences [ ] Behavioural & social sciences [ ] Ecological, evolutionary & environmental sciences

For a reference copy of the document with all sections, see nature.com/documents/nr-reporting-summary-flat.pdf

Life sciences study design

All studies must disclose on these points even when the disclosure is negative.

Sample size	No statistical method was used to predetermine sample size.
Data exclusions	In some animal experiments, one or two samples with extreme deviations from the same group were excluded from data analysis.
Replication	All the experiments were repeated twice or more, and reproducibility was confirmed.
Randomization	All the experiments were performed in a randomized manner.
Blinding	Diarrhea occurrence after allergen challenge was assessed by blinded observers. In other experiments, the investigators was not blinded since randomization minimized their influence on experiments.

Reporting for specific materials, systems and methods

We require information from authors about some types of materials, experimental systems and methods used in many studies. Here, indicate whether each material, system or method listed is relevant to your study. If you are not sure if a list item applies to your research, read the appropriate section before selecting a response.

Materials & experimental systems

n/a	Involved in the study	
[ ]	[x]	Antibodies
[ ]	[x]	Eukaryotic cell lines
[x]	[ ]	Palaeontology and archaeology
[ ]	[x]	Animals and other organisms
[x]	[ ]	Human research participants
[x]	[ ]	Clinical data
[x]	[ ]	Dual use research of concern

Methods

n/a	Involved in the study	
[x]	[ ]	ChIP-seq
[ ]	[x]	Flow cytometry
[x]	[ ]	MRI-based neuroimaging

Antibodies

Antibodies used

FITC-anti-mouse FcεRIα antibody(MAR-1; BioLegend #134306; 1:100 dilution)
Brilliant Violet 421-anti mouse-c-kit antibody (ACK2; BioLegend #135124; 1:100 dilution)
PE-anti-mouse IgE antibody (RME-1; BioLegend #406908; 1:100 dilution)
FITC-anti-mouse CD49b antibody (DX5; BioLegend #108906; :100 dilution)
Anti-mouse FcεR1α antibody (MAR-1; Invitrogen #14-5898-82; 0.5 ug/mL)
Anti-human IgE antibody (RM122; Thermo #SA5-10201; 1:2000 dilution)
Anti-rabbit IgG antibody (Polyclonal; Novus #NB7156; 1:2000 dilution)
HRP-conjugated anti-human IgG4 pFc antibody (HP6023; Southern Biotech #9190-05; 1:10000 dilution)
HRP-conjugated goat anti-mouse IgE antibody (Polyclonal; Southern biotech #1110-05 ; 1:10000 dilution)
Anti-dinitropheyl (DNP) mouse IgE antibody (SPE-7; Sigma-Aldrich #D8406; 20 ug/head)
Anti-NP chimeric human IgE antibody (JW8/1; GeneTax #GTX17414; 1:3 dilution)

Validation

1) FITC-anti-mouse FcεRIα (Verified reactivity, Mouse; For Flow Cytometry analysis)
https://www.biolegend.com/en-us/products/fitc-anti-mouse-fcepsilonrialpha-antibody-5949
2) Brilliant Violet 421-anti mouse-c-kit (Verified reactivity, Mouse; For Flow Cytometry analysis)
https://www.biolegend.com/en-us/products/brilliant-violet-421-anti-mouse-cd117-c-kit-antibody-8637
3) PE-anti-mouse IgE (Verified reactivity, Mouse; For Flow Cytometry analysis)
https://www.biolegend.com/en-us/products/pe-anti-mouse-ige-3267
4) FITC-anti-mouse CD49b (Verified reactivity, Mouse; For Flow Cytometry analysis)
https://www.biolegend.com/en-us/products/fitc-anti-mouse-cd49b-pan-nk-cells-antibody-233

Eukaryotic cell lines

Policy information about cell lines

Cell line source(s)	CHO DG44 cell line was originated from Cricetulus griseus (Chinese hamster) and purchased from Thermo Fischer.<br>LAD-2 cell line used in this study was originated from Homo sapiens (Human, male) and kindly provided by Dean D. Metcalfe (M.D.) of National Institute of Allergy and Infectious Diseases (NIAID).<br>NK103 cell line (FcγRIIIA expressing NK101 cell line) was originated from Homo sapiens (Human, male) and kindly provided by Dr. Sae Won Kim (SL-GIBEN Inc.)
Authentication	None.
Mycoplasma contamination	The LAD2 cell line was not tested for mycoplasma contamination.<br>The CHO DG44 and NK103 cell line was certified as mycoplasma-free.
Commonly misidentified lines (See ICLAC register)	None.

Animals and other organisms

Policy information about studies involving animals; ARRIVE guidelines recommended for reporting animal research

Laboratory animals	BALB/C (Female, 6- to 8-week-old), C3H/HeJ (Female, 4-week-old), Cynomolgus monkey (Female, 39- to 43-month-old)
Wild animals	The study did not include wild animals.
Field-collected samples	The study did not include samples collected from the field.
Ethics oversight	Animal experiments were approved by the Institutional Animal Care and Use Committees (IACUC) of Genexine Inc., CN-Biologics Inc., GI-Biome Inc., Korea Research Institute of Bioscience & Biotechnology and Seoul National University

Note that full information on the approval of the study protocol must also be provided in the manuscript.

Flow Cytometry

Plots

Confirm that:

- [x] The axis labels state the marker and fluorochrome used (e.g. CD4-FITC).
- [x] The axis scales are clearly visible. Include numbers along axes only for bottom left plot of group (a 'group' is an analysis of identical markers).
- [x] All plots are contour plots with outliers or pseudocolor plots.
- [x] A numerical value for number of cells or percentage (with statistics) is provided.

Methodology

Sample preparation	For intestinal mast cell analysis, lamina propria cells of the small intestine were isolated through digestion by collagenase D and DNaseI, and enrichment by 40/75 % percoll density gradient centrifugation.<br>For blood basophil analysis, blood samples were used after removal of red blood cells by ACK lysing buffer.
Instrument	BD LSR Fortessa
Software	Data were acquired using BD FACS DIVA software and analyzed using Flowjo software.
Cell population abundance	Mast cells from Lamina Propria cells : 4~25 %<br>Basophils from PBMC: 0.4~1.0 %
Gating strategy	For Mast cell identification, double positive cells for FceRIa and c-kit was gated after FSC/SSC single cell gating.<br>For basophil identification, double positive cells for IgE and CD49b was gated after FSC/SSC single cell gating.

- [x] Tick this box to confirm that a figure exemplifying the gating strategy is provided in the Supplementary Information.
